# Chloroquine Attenuates Asthma Development by Restoring Airway Smooth Muscle Cell Phenotype *Via* the ROS-AKT Pathway

**DOI:** 10.3389/fphar.2022.916508

**Published:** 2022-06-01

**Authors:** Yan Ren, Xiuhua Zhong, Hongyu Wang, Zhongqi Chen, Yanan Liu, Xiaoning Zeng, Yuan Ma

**Affiliations:** ^1^ Department of Respiratory and Critical Care Medicine, The First Affiliated Hospital of Nanjing Medical University, Nanjing, China; ^2^ Department of Medical Genetics, Nanjing University School of Medicine, Nanjing, China; ^3^ Department of Respiratory and Critical Care Medicine, Affiliated Hospital of Xuzhou Medical University, Xuzhou, China

**Keywords:** chloroquine, airway smooth muscle (ASM) cells, bitter taste receptors (TAS2Rs), TGF-β1, asthma

## Abstract

Switching of airway smooth muscle (ASM) cell phenotype from differentiated-contractile to dedifferentiated-proliferative/synthetic state often occurs in asthmatic subjects with airway dysfunction. Evidence has been provided that chloroquine (an agonist of bitter taste receptors) presented benefits to ASM cell function implicated in asthma. However, the underlying mechanism is unclear. House dust mite (HDM)-sensitized mice were administered with chloroquine or dexamethasone before challenge. BALF and lung tissue were obtained for cell counting, histological analysis or ELISA. Primary cultured ASM cells were stimulated with transforming growth factor (TGF)-β1 or H_2_O_2_. Cells and supernatant were collected for the detection of ASM phenotype, ROS level, and proinflammatory cytokine production. In HDM-sensitized mice, chloroquine attenuated airway hyperresponsiveness (AHR), inflammation and remodeling with an inhibition of immunoglobulin E, IL-4/-13, and TGF-β1 in BALF. ASM cell proliferation (PCNA), hypertrophy (α-SMA), and parasecretion (MMP-9 and MMP-13) were strongly suppressed by chloroquine, hinting the rebalance of the heterogeneous ASM populations in asthmatic airway. Our data *in vitro* indicated that chloroquine markedly restrained maladaptive alteration in ASM phenotype in concert with a remission of ROS. Using H_2_O_2_ and PI3K inhibitor (LY294002), we found that the inhibition of oxidative stress level and ROS-AKT signal by chloroquine may serve as a potential mechanism that dedicates to the restoration of the phenotypic imbalance in ASM cells. Overall, the present findings suggested that chloroquine improves asthmatic airway function by controlling ASM cell phenotype shift, sketching a novel profile of chloroquine as a new therapeutic candidate for airway remodeling.

## Introduction

As the major characteristic of asthma (Al-Muhsen et al., 2011), airway remodeling encompasses a range of events such as alterations in airway smooth muscle (ASM), which definitively determine the severity and outcome of the disease (Zhang and Li, 2011). ASM cells retain a remarkable phenotypic plasticity. Insults such as infection, allergens and environmental factors can alter the profile of ASM cells from a differentiated and quiescent “contractile” state to a dedifferentiated and proliferative “synthetic” state (Liu et al., 2015). The “synthetic” ASM cells involve features such as cell hypertrophy, hyperplasia and parasecretion, which contribute to the persistent AHR, irreversible airway obstruction, and exacerbation of inflammation. Hence, a “vicious cycle” occurs. Many strategies have been developed for asthma management, but few proved effective in stopping “the remodeling clock”. Therefore, an urgent need for the development of new therapeutic options targeting airway remodeling has been brought into schedule.

Bitter taste receptors (TAS2Rs) were originally found to be expressed in tongue, responsible for our perception of bitter taste. However, they are also found recently in other tissues including ASM cells which might play a key role in airway remodeling. An upregulation of TAS2Rs was detected in those children with severe asthma. And inhaled bitter tastants helped to decrease the obstruction of asthmatic airway (Deshpande et al., 2010; Pulkkinen et al., 2012). Activation of TAS2Rs by chloroquine and denatonium has been proved to be able to cause the relaxation of ASM (Deshpande et al., 2010; Pulkkinen et al., 2012) and inhibit the secretion of proinflammatory cytokines (Orsmark-Pietras et al., 2013). All these findings suggest TAS2Rs as the potential targets for the management of ASM cell function and open up new avenues for asthma treatment.

**FIGURE 1:**
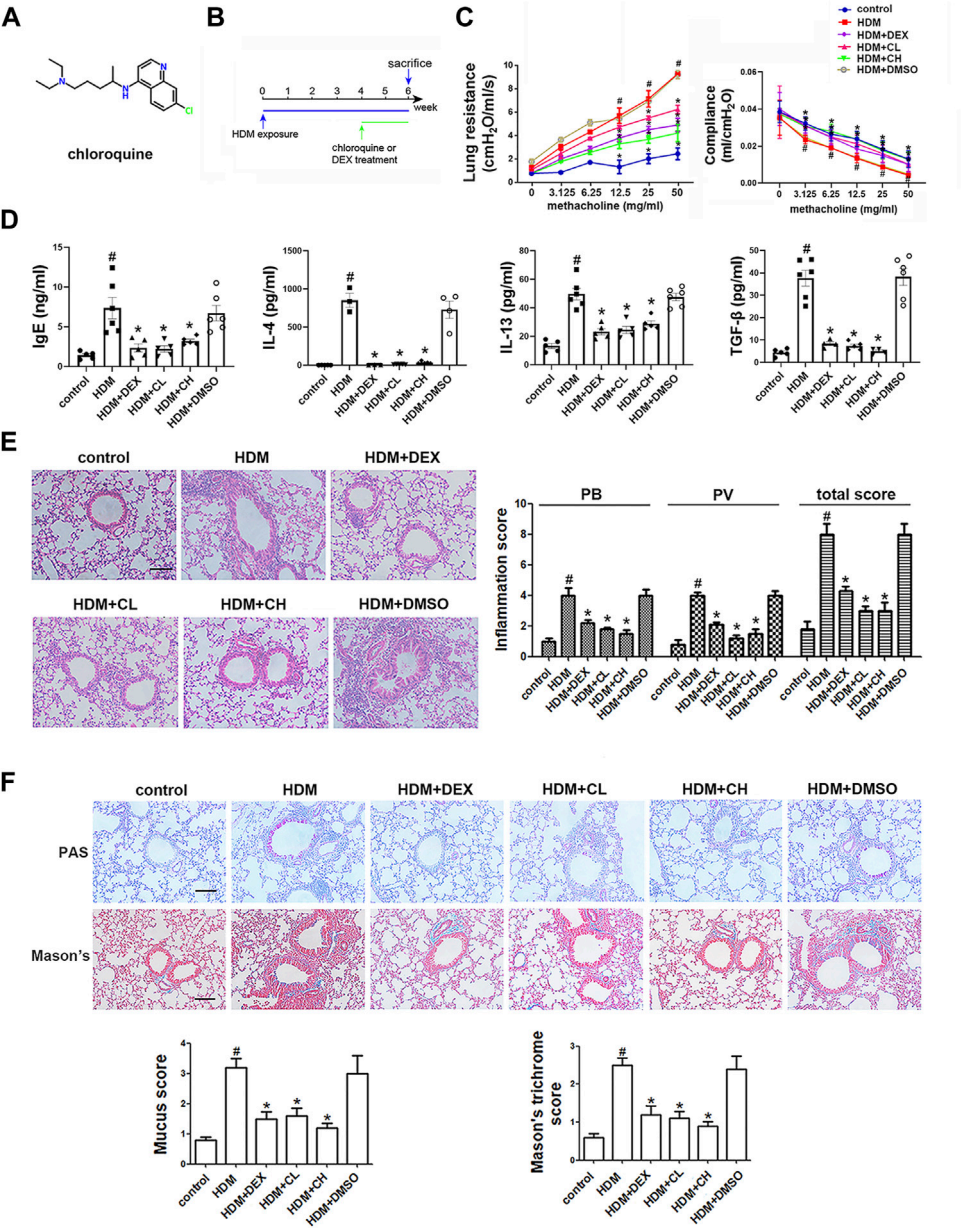
Chemical structure of chloroquine and experimental protocol for the chronic asthma model. Treatment with chloroquine alleviated AHR, ameliorated lung dynamic compliance, decreased the levels of IgE, Th2 cytokines, and TGF-β1 in BALF, and inhibited inflammatory cell infiltration, goblet cell hyperplasia, collagen deposition in lung **(A)** Chemical structure of chloroquine **(B)** Brief scheme of HDM sensitization and challenge **(C)** Increasing inhaled doses of methacholine (3.125–50 mg/ml), lung resistance and dynamic compliance in mice **(D)** The concentrations of HDM-specific IgE, IL-4, IL-13, and TGF-β1 were measured by ELISA **(E)** Lung sections were stained with H&E to analyze the infiltration of inflammatory cells (magnification, ×400; scale bar, 50 µm). Layers of inflammatory cells were counted, and the total inflammation score was summed with the peribronchial (PB) and perivascular (PV) inflammation scores **(F)** Lung sections were stained with PAS to assess goblet cell hyperplasia and Masson’s trichrome to evaluate the subepithelial deposition of collagen and fibrosis (magnification, ×400; scale bar, 50 µm). PAS-positive and PAS-negative epithelial cells were counted, and the percentage of PAS-positive cells per bronchiole was calculated. Masson’s trichrome staining analysis of collagen deposition was calculated. Values are the mean ± SEM (*n* = 6 per group). #*p* < 0.05 compared with the control group, and *p < 0.05 compared with the HDM group.

Chloroquine (N4-(7-chloroquineolin-4-yl)-1N,N1-diethylpentane-1,4-diamine) (Figure 1A) has been proposed as a classic TAS2Rs agonist in digestive system and respiratory system. It was revealed that chloroquine could lead to relaxation of precontracted airways (Becker et al., 1984; Colacone et al., 1990; Kivity et al., 1990; Grassin-Delyle et al., 2015), as well as inhibition of ASM cell growth and cytokine production (Orsmark-Pietras et al., 2013; Ekoff et al., 2014). Although TAS2Rs have got growing attention in respiratory diseases, the actual effects of chloroquine on ASM cells and the precise mechanism involved in asthma have not been clarified.

In this study, the house dust mite (HDM) model was established to illustrate the impacts of chloroquine during asthma progressing. With an emphasis on the modulation of cellular phenotypes, an *in vitro* model of human ASM cells stimulated with TGF-β1 was used to elucidate the underlying mechanism involved.

## Materials and Methods

### Animals

Thirty-six specific pathogen-free female BALB/c mice (18–22 g) aged 6–8 weeks were obtained from the Animal Core Facility of Nanjing Medical University (Nanjing, China). The mice were maintained in a temperature-controlled room under a 12-h dark/12-h light cycle and provided food and water. All experiments involving animal and tissue samples were performed in accordance with the guidelines established by the National Institutes of Health and Nanjing Medical University, and all of the procedures were approved by the Institutional Animal Care and Use Committee of Nanjing Medical University (Nanjing, China). The BALB/c mice were randomly divided into the six groups: control group; HDM group, treated with HDM (Greer Laboratories, Lenoir, NC, United States); HDM + CL group, treated with HDM and 1 mg/kg chloroquine (Sigma-Aldrich, St. Louis, MO, United States); HDM + CH group, treated with HDM and 5 mg/kg chloroquine; HDM + dexamethasone group, treated with HDM and 1 mg/kg DEX (Sigma-Aldrich); and HDM + DMSO group, treated with HDM and 0.5% dimethyl sulfoxide (DMSO, Sigma-Aldrich). The mice received intranasal droplets containing the purified HDM extract [25 μg of protein solubilized in 25 μL of phosphate-buffered saline (PBS)] for 5 days/week for up to six consecutive weeks. During the last 2 weeks of the HDM treatment, the mice were intraperitoneally administered chloroquine, DEX or vehicle (DMSO) 1 h prior to the HDM challenge. All treatments were administered under isoflurane anesthesia and ended 24 h before sacrifice. Bronchoalveolar lavage fluid (BALF) and lung tissues were collected for analyses (Figure 1B).

### Measurement and Analysis of Airway Responsiveness

The lung function was evaluated from direct measurements of lung resistance and dynamic compliance in restrained, tracheostomized, mechanically ventilated mice using the FinePointe RC system (Buxco Research Systems, Wilmington, NC, United States) under general anesthesia as described previously (Kerzerho et al., 2013).

### BALF Collection and Test

The tracheae were exposed, and BALF was collected *via* lavage with ice-cold PBS (400 μL × 3; 85–90% of the lavage volume was recovered) using a tracheal catheter. The lavage samples from each mouse were centrifuged at 1000 rpm at 4°C for 10 min. The supernatant was collected, divided into four equal portions and frozen at −80°C for enzyme-linked immunosorbent assay (ELISA).

### Lung Histology

BALF samples were obtained, and the left lung was immersed in 4% paraformaldehyde and then embedded in paraffin. A series of microsections (5 μm) were cut using a microtome and stained with hematoxylin and eosin (H&E) for assessments of inflammatory cell infiltration. An inflammation score was determined as follows: grade 0, no inflammation; grade 1, occasional cuffing with inflammatory cells; and grades 2, 3, and 4, most bronchi or vessels surrounded by a thin layer (1–2 cells), a moderate layer (3–5 cells), or a thick layer (>5 cells) of inflammatory cells, respectively. The total inflammation score was calculated by the addition of the peribronchial (PB) and perivascular (PV) inflammation scores. Periodic acid-Schiff (PAS) staining was used for the quantification of airway goblet cells, and Masson’s trichrome staining was used for the visualization of collagen deposition and fibrosis. Both staining methods were scored as follows: 0, none; 1, <25%; 2, 25–50%; 3, 50–75%; and 4, >75% goblet cells (Ma et al., 2016). The sections were also immunohistochemically stained for matrix metalloproteinase (MMP)-9, MMP-13, proliferating cell nuclear antigen (PCNA) and alpha-smooth muscle actin (α-SMA). For the semiquantitative evaluation of the expression of MMP-9, MMP-13, PCNA and α-SMA, the IOD of the results was analyzed using Image-Pro Plus 6.0 software. The mean percentages of positive epithelial cells in the bronchi were determined in at least five areas at ×400 magnification and assigned to one of the following categories: 0, <5%; 1, 5–25%; 2, 25–50%; 3, 50–75%; and 4, >75% (Ma et al., 2016). The immunostaining intensities of MMP-9, MMP-13, PCNA and α-SMA were scored as 1+ (weak), 2+ (moderate) or 3+ (intense). The percentage of positive epithelial cells and the staining intensities were multiplied to yield a weighted score for each case. Two independent observers who were blinded to the experiment calculated all of the scores, and at least three different fields of each lung section were examined.

### *In situ* Detection of ROS

The *in situ* production of ROS in frozen sections was evaluated microscopically using dihydroethidium (DHE) (Beyotime Institute of Biotechnology, Jinan, Shandong, China) (Boldogh et al., 2005). Briefly, snap-frozen lung tissue samples were embedded in Tissue-Tek OCT compound, cryosectioned at 7 mm, allowed to air-dry at room temperature, and stored at -80°C until needed. The slides were placed in PBS for 30 min at room temperature and stained with DHE (10 mm) in PBS for 30 min in a moist chamber in the dark. The slides were rinsed extensively with PBS, covered with a coverslip, and imaged using a fluorescence microscope (Zeiss LSM 5 LIVE, Germany). The DHE fluorescence was quantified by averaging the mean value of the fluorescence intensity using Image-Pro Plus 6.0. Four sections from each mouse were analyzed using this procedure, and the mean value of the fluorescence was calculated.

### Culture and Treatment of Normal Human ASM Cells

Normal human ASM cells were purchased from ScienCell Research Laboratories (Carlsbad, CA, United States) and cultured at 37°C in the presence of 5% CO_2_ in smooth muscle cell medium (ScienCell) supplemented with 20 U/l penicillin, 20 μg/ml streptomycin, 1% smooth muscle cell growth supplement and 2% fetal bovine serum (ScienCell). Cells between passages four and eight were used for the experiments. After serum starvation for 6–8 h (Supplementary Figure S1), the ASM cells were stimulated with 5 ng/ml TGF-β1 (Peprotech) or H_2_O_2_ (20 μm, Sigma-Aldrich) alone or in combination with chloroquine (10 μm) or N-acetylcysteine (NAC, 10 mm, Sigma-Aldrich) or LY294002 (10 μm, Sigma-Aldrich). The cells were further cultured for the indicated durations.

### Lactate Dehydrogenase Release Assay

LDH assay (Jiancheng, Nanjing, China) was adopted to determine the cytotoxicity of chloroquine in ASM cells. As previously described, ASM cells were planted at a density of 5×10^3^ per well in a 96-well plate and treated with chloroquine for 48 h. Then the supernatant was centrifuged and transferred to be mixed with matrix buffer and coenzyme I application solution. After incubation with 2,4-dinitrophenylhydrazine, 0.4 M NaOH was added into the system to terminate the reaction. The release of LDH from ASM cells was then evaluated by colorimetric assay with a microplate reader (CANY, Shanghai, China) at 450 nm. Each sample was tested in triplicate.

### Cell Viability Assay

The proliferation of ASM cells was determined using cell counting kits (CCK)-8 and 5-ethynyl-2′-deoxyuridine (EdU) assays. The ASM cells were cultured in a 96-well plate at a density of 5×10^3^ cells per well and treated with TGF-β1 and chloroquine at concentrations from 0.1 to 100 μm for 48 h. The CCK-8 solution (Dojindo Molecular Technologies, Inc. Kumamoto, Japan) was added to the cell culture medium at a dilution of 1:10, and the cultures were incubated for another 1–2 h at 37°C. The absorbance at 450 nm (A450) was measured using a microplate reader (CANY, Shanghai, China). For the EdU assay, the ASM cells were cultured using the aforementioned procedure. After 48 h of treatment, the cells were labelled using an EdU assay kit (Ribobio, Guangzhou, China) according to the manufacturer’s instructions. Images were obtained using a fluorescence microscope (Olympus IX71, Japan). Each sample was measured in triplicate.

### Transmission Electron Microscopy

ASM cell phenotype was observed using TEM. The ASM cell culture medium was discarded, and the cells were fixed with an electron microscope fixative solution at 4°C for 2 h. The ASM cells were centrifuged at low speed, wrapped with 1% agarose, and rinsed with 0.1 M PBS. The cells were fixed with 1% osmium acid at room temperature for 2 h, rinsed with 0.1 M PBS, and dehydrated using an alcohol and acetone gradient for 15 min. The mixture was subsequently combined with acetone and the embedding agent at a ratio of 1:1, infiltrated for 2–4 h, mixed with acetone and the embedding agent at a ratio of 1:2, infiltrated overnight, treated with the pure embedding agent for 5–8 h, poured into an embedding plate and placed overnight in an oven at 37°C. After penetration, the plate was heated in an oven at 60°C for 48 h for polymerization. Ultrathin slices (60–80 nm) were obtained using a tissue slicer. After double staining with uranium (alcohol solution saturated with 2% uranyl acetate, 15 min) and lead (lead citrate, 15 min), the sections were dried overnight at room temperature and observed and analyzed using TEM.

### Enzyme-Linked Immunosorbent Assay

To examine the effects of chloroquine on airway inflammation and the microenvironment *in vitro*, the levels of interleukin-8 (IL-8), monocyte chemoattractant protein-1 (MCP-1), soluble intercellular adhesion molecule-1 (ICAM-1), vascular endothelial growth factor (VEGF, R&D Systems, Minneapolis, MN, United States) and isoprostane-8 (IP-8) (Senbeijia Corp. Nanjing, China) were measured. The ASM cells were cultured using the aforementioned procedure. After starvation in serum-free medium, the cells were stimulated with TGF-β1 or H_2_O_2_ alone or in combination with chloroquine or NAC for 6–8 h and then subjected to ELISA. The level of HDM-specific immunoglobulin E (IgE, Chondrex, Inc. Redmond, WA, United States) in the serum and the levels of TGF-β1, IL-4, and IL-13 (R&D Systems) in the BALF of the mice were also measured using ELISA according to the manufacturer’s instructions.

### Determination of Malondialdehyde Level

The MDA levels in the cell culture medium were determined using the thiobarbituric acid reacting substances (TBARS) assay (Senbeijia Corp. Nanjing, China) as previously described (Ma et al., 2016). MDA reacts with thiobarbituric acid under acidic conditions at 95°C to form a pink-colored complex that can be measured at 532 nm, and 1,3,3-tetra ethoxy propane (TEP) was used as a standard.

### Determination of Intracellular ROS Production

The intracellular ROS level was measured using the 2′,7′-dichlorofluorescin diacetate (DCFH-DA, Sigma-Aldrich) assay. Briefly, 1.5×10^4^ ASM cells were seeded into each well of a six-well plate and cultured for 24 h. The cells were then divided into four groups: control group; TGF-β group, treated with 5 ng/ml TGF-β1; TGF-β+C group, treated with 5 ng/ml TGF-β1 and 5 μm chloroquine; and C group, treated with 5 μm chloroquine. The cells were then cultured for an additional 24 h and incubated with 10 μm DCFH-DA for 30 min at 37°C in the dark. The cells were subsequently washed twice with PBS and analyzed within 30 min using a FACScan instrument (Becton Dickinson, San Jose, CA, United States) with an excitation setting of 488 nm. The specific fluorescence signals corresponding to DCFH-DA were determined using a 525-nm bandpass filter.

### Western Blot Analysis

The cells were homogenized and lysed in RIPA buffer (Sigma-Aldrich) supplemented with a protease inhibitor and a phosphatase inhibitor (Selleck). Equal amounts of proteins were separated using 10% SDS-PAGE. After electrophoresis, the separated proteins were transferred to polyvinylidene difluoride membranes (Millipore, Billerica, MA, United States) using the wet transfer method. Nonspecific sites were blocked with 5% nonfat milk in TBS Tween 20 [TBST; 25 mm Tris (pH 7.5), 150 mm NaCl, and 0.1% Tween 20] for 2 h, and the blots were incubated with primary antibodies (Cell Signaling Technology, Inc.), including β-actin, anti-phospho-protein kinase B (AKT), anti-AKT, α-SMA, fibronectin and collagen I antibodies overnight at 4°C. Goat anti-rabbit horseradish peroxidase-conjugated IgG (Cell Signaling Technology, Inc.) was used for the detection of antibody binding. The membranes were treated with enhanced chemiluminescence system reagents (Thermo), and the binding of specific antibodies was visualized using a Bio-Rad Gel Doc/ChemiDoc Imaging System and analyzed using Quantity One software.

### Statistical Analysis

The data are expressed as the means ± standard errors of the mean (SEM). All of the tests were performed using Prism 6.00 (GraphPad Software, San Diego, CA, United States) and SPSS version 20 (SPSS, Inc. Chicago, IL, United States). To determine the differences between multiple groups, the results were analyzed using one-way analysis of variance for repeated measures followed by Dunnett’s post hoc test. The significance level was set to *p* < 0.05.

## Results

### Chloroquine Relieves AHR and HDM-specific IgE

In this study, HDM was used to establish a mouse model of asthma, and chloroquine-mediated alleviation of asthma was examined (Figure 1B; Supplementary Figure S2). Our results showed that chloroquine protected lungs from HDM-induced AHR (as assessed by reduced lung resistance) and preserved lung function (as assessed by increased dynamic compliance) (Figure 1C; Supplementary Figure S3; Supplementary Video S1). A high level of IgE is well established as a feature of asthma. After the sensitization and challenge, HDM-specific IgE level in the BALF was significantly elevated in the HDM and HDM + DMSO groups, and the administration of chloroquine and DEX abolished this increase (Figure 1D). These findings indicated that the asthma model was successful and chloroquine alleviated AHR and HDM-specific IgE level.

### Chloroquine Inhibits Airway Inflammation, Goblet Cell Proliferation and Collagen Deposition/Fibrosis

Extensive infiltration of inflammatory cells was observed around the respiratory tracts and vessels in the HDM and HDM + DMSO groups (Figure 1E), which indicated that the HDM and HDM + DMSO groups exhibited more severe airway inflammatory responses than the control group. Similarly, airway challenge with HDM notably increased IL-4 and IL-13 levels in the BALF. The administration of chloroquine dose-dependently reduced the IL-4 and IL-13 levels in the BALF compared to the levels in the HDM group (Figure 1D). To evaluate the extent of goblet cell and mucus production, the lung sections were stained with PAS, and the percentage of PAS-positive cells in the airway epithelium was determined. We found that the HDM-challenged mice developed marked goblet cell hyperplasia and mucus hypersecretion in the lumen of the bronchioles (Figure 1F). The mice treated with DEX and high doses of chloroquine had fewer goblet cells in the airway epithelium and reduced mucus scores of 1.5 ± 0.24 and 1.2 ± 0.15 (*p* < 0.05), respectively. The area of collagen deposition/fibrosis was assessed using Masson’s trichrome staining, and this analysis revealed that the extent of collagen deposition/fibrosis was profoundly enhanced over the interstitia of the airways and vessels of tissues of the HDM-challenged mice compared to the control mice (Figure 1F). The administration of 5 mg/kg chloroquine significantly ameliorated these serious pathophysiological changes, which resulted in a score of 1.1 ± 0.12 (*p* < 0.05), and modest effects were observed with the administration of DEX and low doses of chloroquine. These findings indicated that chloroquine inhibited airway inflammation, goblet cell proliferation and collagen deposition/fibrosis in the HDM-induced asthma model.

### Chloroquine Decreases the Levels of Proliferation-Associated Proteins and ROS *in vivo*

**FIGURE 2:**
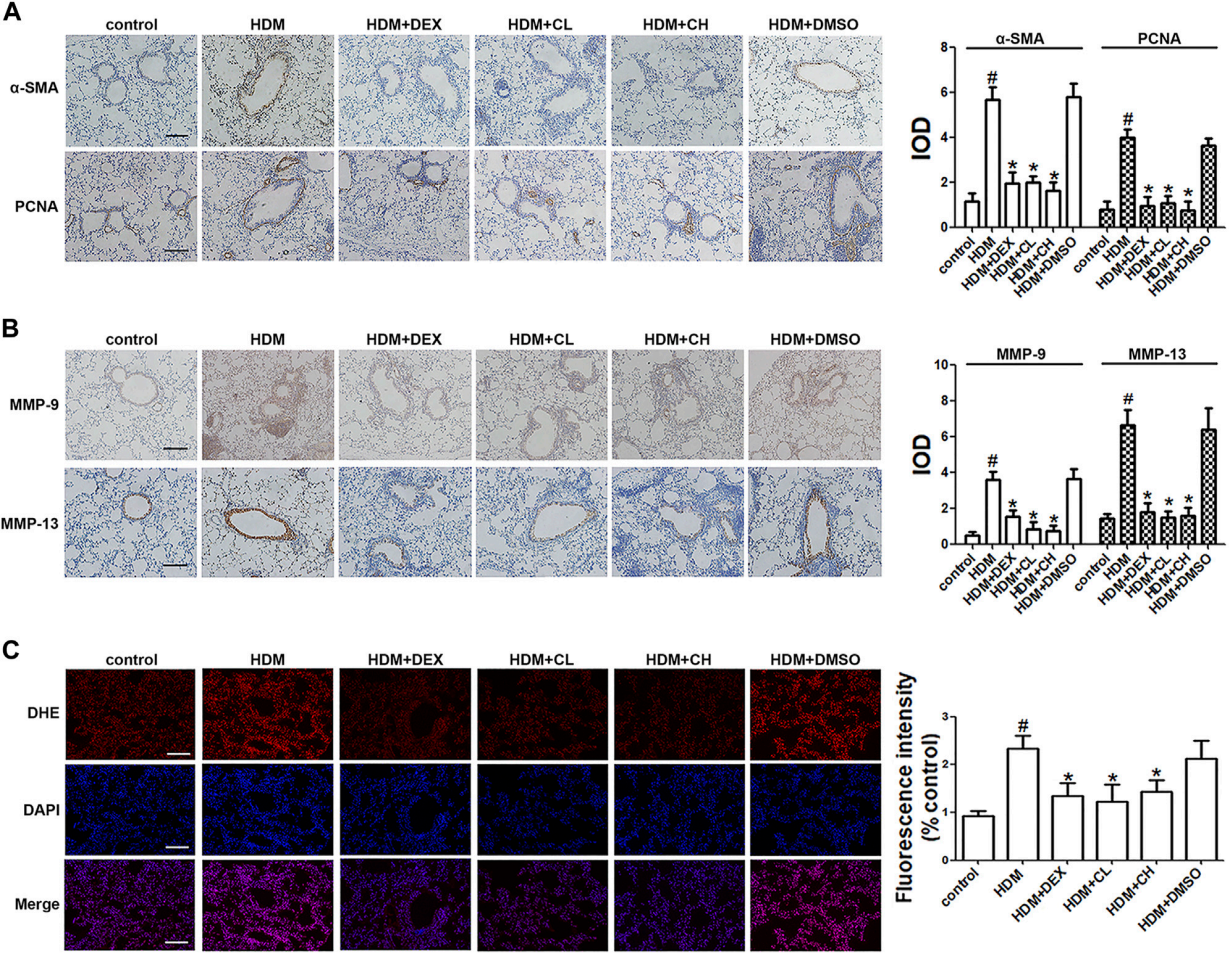
Treatment with chloroquine inhibited the expression of α-SMA, PCNA, MMP-9, MMP-13, and in situ ROS levels in lung tissue **(A,B)** Immunohistochemical staining was performed to assess the distribution of α-SMA, PCNA, MMP-9, and MMP-13 (magnification, ×400; scale bar, 50 µm), and their expression was evaluated, respectively. The staining scores were multiplied by the percentage of positive epithelial cells and staining intensity scores **(C)** Frozen lung sections were stained with DHE, imaged and analyzed by Image-Pro Plus 6.0. The fluorescence values were expressed as the ratio to the levels in the control cells. Values are the mean ± SEM (*n* = 6 per group). #*p* < 0.05 compared with the control group, and *p < 0.05 compared with the HDM group.

As a phenotypic marker of ASM cells, a-SMA was detected using immunohistochemical techniques. The α-SMA staining densities in the ASM of the HDM-challenged mice were higher than the control mice (*p* < 0.05). As shown in Figure 2A, the administration of chloroquine markedly decreased the α-SMA-stained smooth muscle layer. Treatment with DEX also decreased the α-SMA staining densities, but these effects were less obvious than the chloroquine effects. The immunohistochemistry assays also showed stronger proliferation-associated protein PCNA, MMP-9 and MMP-13 staining around the bronchioles and in the infiltrated inflammatory cells in the HDM-challenged mice compared to the control mice. The administration of DEX or high doses of chloroquine markedly reversed these increases (Figures 2A,B). To determine whether chloroquine inhibited HDM-induced airway inflammation and remodeling *via* the scavenging of free radicals, we detected the *in situ* ROS level in the lung tissue to evaluate the changes resulting from HDM-induced oxidative damage. As shown in Figure 2C, DHE staining was used to assess the *in situ* ROS level in the lungs. DHE was predominately detected in the airway epithelium in control mice. Challenge with HDM enhanced the fluorescence not only in the inflammatory cells and epithelium, but also ASM cells around the airway, whereas treatment with chloroquine and DEX weakened the fluorescence in those cells of mice. TGF-β1 is a key mediator in asthmatic airway remodeling. After sensitization and challenge, the TGF-β1 level in the BALF was also upregulated in the HDM and HDM + DMSO groups compared to the control group (*p* < 0.05). Chloroquine and DEX almost reversed this upregulation (Figure 1D). These observations suggested that the protective effect of chloroquine was attributed to the downregulation of TGF-β1.

### Chloroquine Suppresses TGF-β1-Induced ROS *in vitro*

**FIGURE 3:**
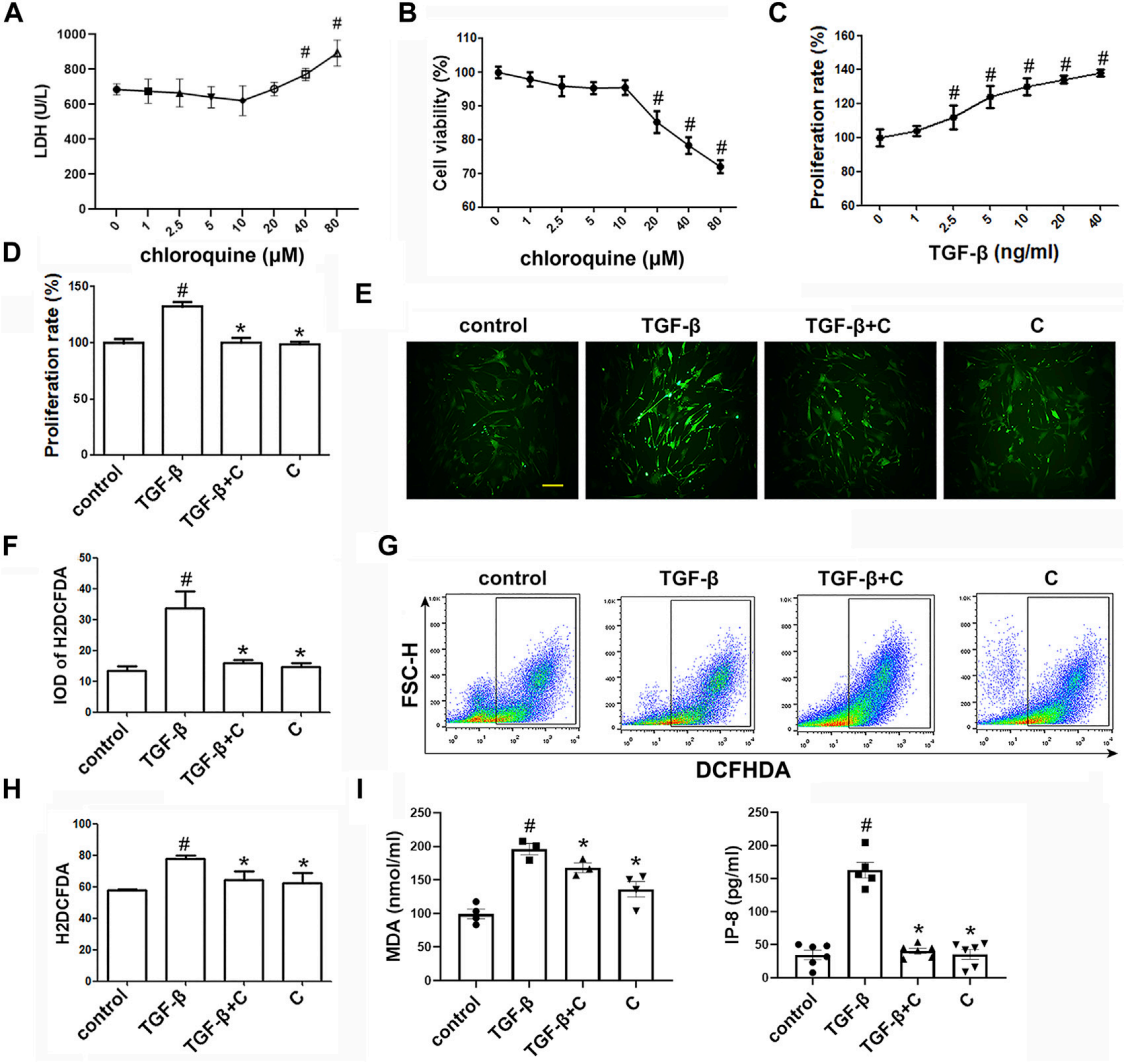
Treatment with chloroquine inhibited ASM cell proliferation, ROS generation and MDA/IP-8 production elicited by TGF-β1 **(A)** Cytotoxicity of chloroquine in human ASM cells (LDH assay) **(B)** Cell viability with diverse concentrations of chloroquine (CCK-8 assay) **(C)** Cell proliferation provoked by TGF-β1 (CCK-8 assay) **(D)** Inhibition of TGF-β1-stimulated cell proliferation by chloroquine **(E,F)** DCFH-DA fluorescence (green) imaging of ROS with a laser scanning confocal microscope (magnification, ×400; scale bar, 50 µm) **(G,H)** Fluorescence-activated cell sorting profile of ROS with DCFH-DA by flow cytometry **(I)** Analysis for MDA and IP-8 content in supernatant. Values are the mean ± SEM of at least four independent experiments performed in triplicate. #*p* < 0.05 compared with the control, and *p < 0.05 compared with the TGF-β1 group. C, chloroquine (5 μm); TGF-β, TGF-β1 (5 ng/ml).

The toxicity of chloroquine (1, 2.5, 5, 10, 20, 40, and 80 μm) on human ASM cells as shown in Figure 3A, and the cell viability after 48 h of treatment with 5 μm chloroquine was 97 ± 3% (Figure 3B). Chloroquine decreased the proliferation of ASM cells *in vitro* in a dose-dependent manner, and the proliferation of human ASM cells was enhanced after TGF-β1 stimulation. The concentration of TGF-β1 (5 ng/ml) induced significant ASM cell proliferation, as determined by the CCK-8 assays, after 48 h of treatment (Figure 3C). Treatment with 5 μm chloroquine markedly decreased TGF-β1-induced ASM cell proliferation from 132 ± 3%–104 ± 2% (*p* < 0.05) (Figure 3D). Then, we used 5 μm chloroquine for further study and determined whether ROS were involved in TGF-β1-induced proliferation. As shown in Figure 3E, treatment with TGF-β1 activated ROS. A H2DCFDA analysis was performed to evaluate the level of intracellular ROS in ASM cells (Figure 3F). The results suggested that treatment of ASM cells with chloroquine significantly reduced the TGF-β1-induced fluorescence, which reflects the level of oxidative damage. A flow cytometry analysis showed that treatment with chloroquine decreased the intracellular production of ROS from 79 ± 2%–64 ± 4% (*p* < 0.05) (Figures 3G,H). As shown in Figure 3I, the concentrations of MDA and IP-8 in the TGF-β1-treated group were significantly higher than the control group (*p* < 0.05), and treatment with chloroquine significantly decreased the increased levels of MDA and IP-8 (*p* < 0.05).

### Chloroquine Reverses TGF-β1-Induced Proliferative/Synthetic ASM Cells

**FIGURE 4:**
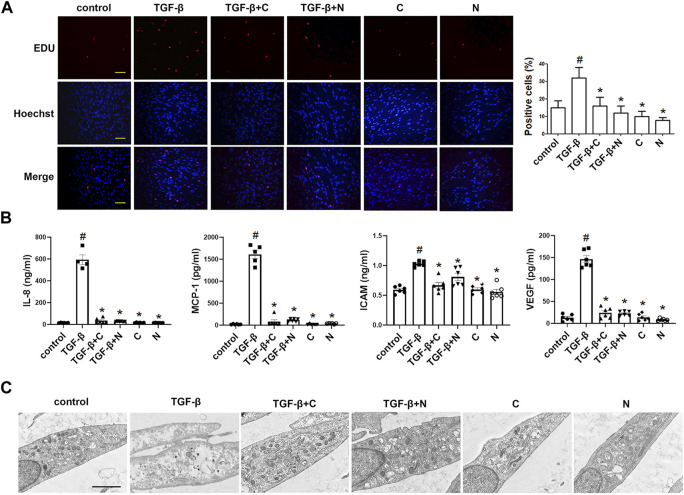
Treatment with chloroquine inhibited TGF-β1-induced proliferation, hypersecretion and hypertrophy in ASM cells **(A)** Effect of chloroquine on TGF-β1-stimulated human ASM cells as assessed by EdU assay (magnification, ×200; scale bar, 100 µm) **(B)** The IL-8, MCP-1, ICAM-1, and VEGF levels in the supernatant were measured by ELISA **(C)** Effect of chloroquine on TGF-β1-stimulated human ASM cells as assessed by transmission electron microscope (TEM) (magnification, ×7000; scale bar, 2 µm). Values are the mean ± SEM of at least four independent experiments performed in triplicate. #*p* < 0.05 compared with the control, and *p < 0.05 compared with the TGF-β1 group. C, chloroquine (5 μm); TGF-β, TGF-β1 (5 ng/ml); N, NAC (10 mm).

We used the EdU incorporation assay, which is a more sensitive and specific method (Yu et al., 2009), to evaluate the effects of chloroquine on TGF-β1-induced cell proliferation. As shown in Figure 4A, TGF-β1 stimulation significantly increased the number of cells that incorporated EdU compared to the control, and treatment with chloroquine or NAC (10 mm) clearly limited the TGF-β1-induced proliferation. As previously mentioned, ASM cells tend to proliferative/synthetic phenotype in asthmatic patients, and release chemokines and adhesion molecules to induce inflammatory responses and stimulate eosinophil migration (Zha et al., 2013). To ascertain the anti-inflammatory mechanism of chloroquine, we studied the effects of chloroquine on the TGF-β1-induced upregulation of IL-8, MCP-1, ICAM-1 and VEGF in human ASM cells. The results showed that chloroquine and NAC substantially blocked the TGF-β1-induced upregulation of IL-8, MCP-1, ICAM-1, and VEGF expression in human ASM cells, respectively (Figure 4B). The TGF-β1-mediated phenotype switching of ASM cells was assessed by TEM. The proliferative/synthetic ASM cells appeared flattened with numerous cytoplasmic processes and an elongated oval nucleus containing little or no heterochromatin. The cytoplasm contained mitochondria, highly developed Golgi cisternae and numerous profiles of rough endoplasmic reticulum. The results showed that chloroquine and NAC also reversed TGF-β1-induced proliferative/synthetic ASM cells (Figure 4C). Based on the above results, we conclude that chloroquine rebalances ASM cell phenotype, and the mechanism may be related to the regulation of ROS.

### Chloroquine Reverses H_2_O_2_-Induced Proliferative/Synthetic ASM Cells

**FIGURE 5:**
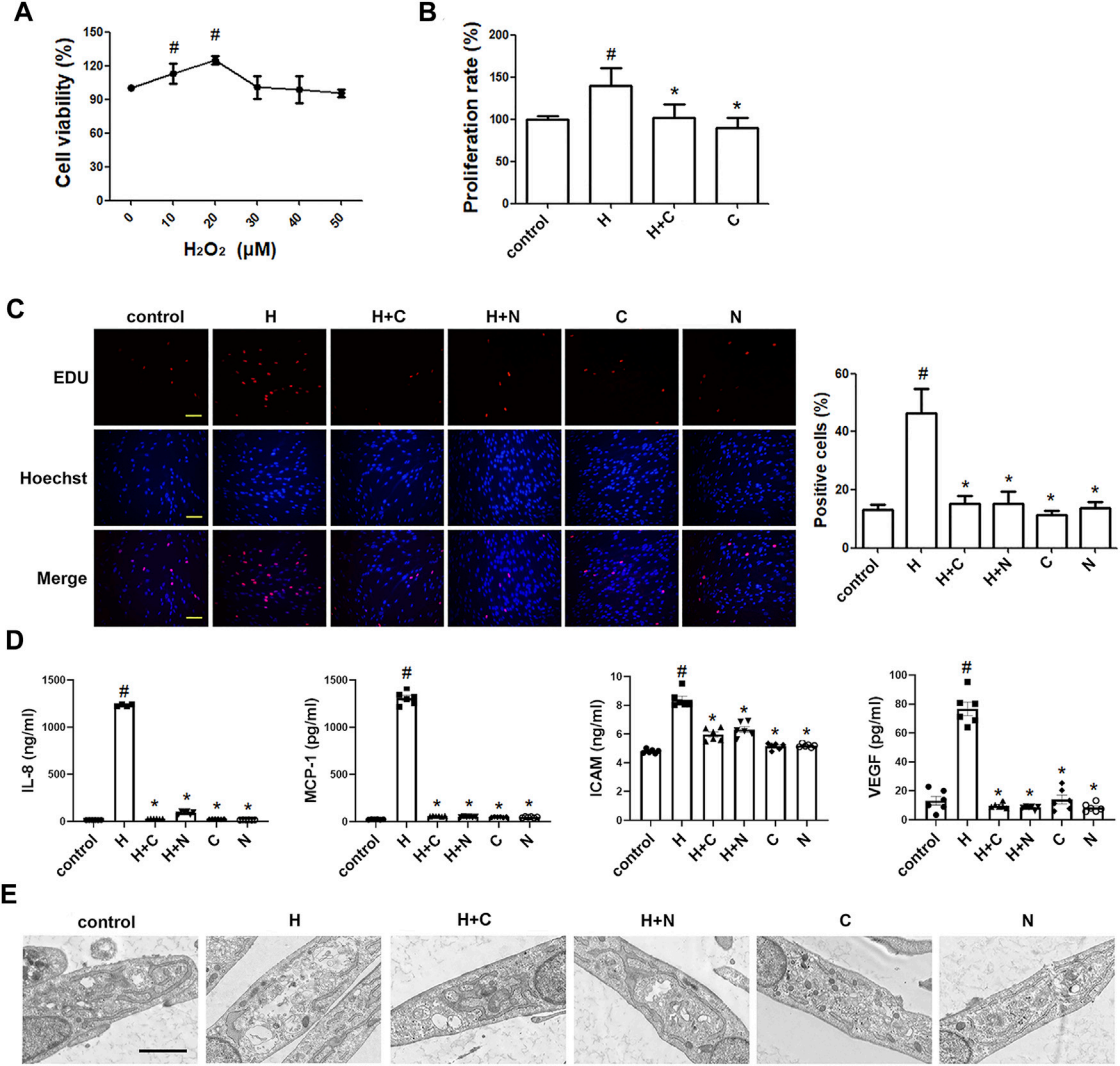
Treatment with chloroquine inhibited the H2O2-induced proliferation, hypersecretion and hypertrophy in ASM cells **(A)** Effect of stimulation with different concentrations of H2O2 on the proliferation of human ASM cells as assessed by CCK-8 assay **(B)** Effect of chloroquine on H2O2-stimulated human ASM cells as assessed by CCK-8 assay **(C)** Effect of chloroquine on H2O2-stimulated human ASM cells as assessed by EdU assay (magnification, ×200; scale bar, 100 µm) **(D)** The IL-8, MCP-1, ICAM-1, and VEGF levels in the supernatant were measured by ELISA **(E)** Effect of chloroquine on H2O2-stimulated human ASM cells as assessed by TEM (magnification, ×7000; scale bar, 2 µm). Values are the mean ± SEM of at least four independent experiments performed in triplicate. #*p* < 0.05 compared with the control, and *p < 0.05 compared with the TGF-β1 group. C, chloroquine (5 μm); H, H2O2 (20 μm); N, NAC (10 mm).

Furthermore, we studied whether oxidative stress involves in the ASM cell phenotype switching. As shown in Figures 5A–C, H_2_O_2_ (20 μm) stimulation directly increased the number of cells compared to the control, and treatment with chloroquine or NAC clearly limited the H_2_O_2_-induced proliferation. Chloroquine and NAC also inhibited H_2_O_2_-induced expression of IL-8, MCP-1, ICAM-1, and VEGF in human ASM cells, respectively (Figure 5D). The TEM results showed that the ASM cell phenotype switching induced by H_2_O_2_ (20 μm) was almost the same as that induced by TGF-β1 (5 ng/ml), and chloroquine also reversed H_2_O_2_-induced proliferative/synthetic ASM cells (Figure 5E). Based on the above results, we conclude that chloroquine rebalances ASM cell phenotype *via* the ROS pathway.

### Chloroquine Rebalances ASM Cell Phenotype *via* the AKT Signaling Pathway

**FIGURE 6:**
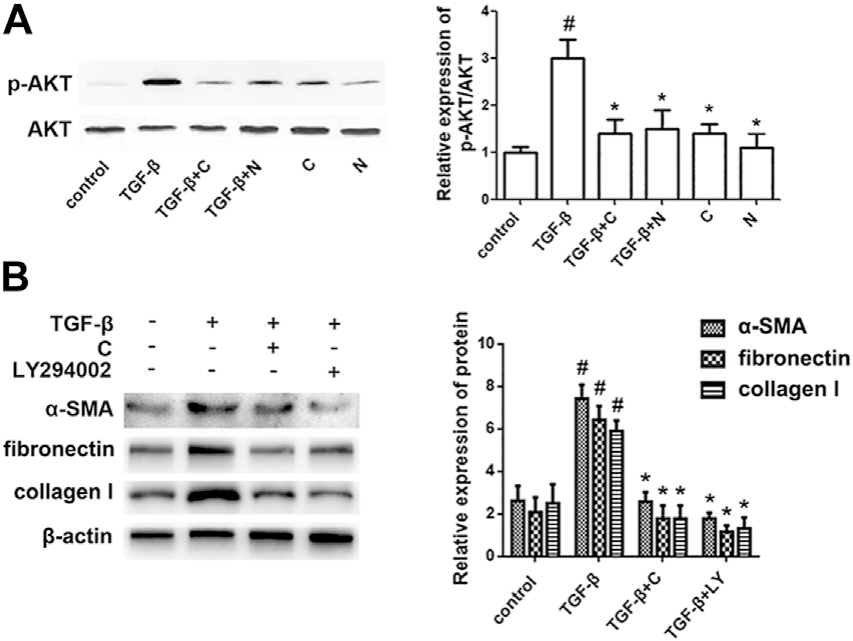
Treatment with chloroquine suppressed the activation of the AKT pathway **(A)** Treatment with chloroquine suppressed the TGF-β1-induced phosphorylation of AKT in ASM cells. The phosphorylation and total protein content of AKT were measured by western blot. The relative density quantification was phosphorylated proteins relative to total proteins **(B)** The contents of α-SMA, fibronectin and collagen I were measured by western blot. Values are the mean ± SEM of at least four independent experiments performed in triplicate. #*p* < 0.05 compared with the control, and *p < 0.05 compared with the TGF-β1 group. C, chloroquine (5 μm); TGF-β, TGF-β1 (5 ng/ml); N, NAC (10 mm); LY, LY294002 (10 μm).

To determine the signaling mechanisms underlying the effects of chloroquine on airway remodeling, the phosphorylation status of AKT was investigated. TGF-β1-induced ASM cells were treated with chloroquine and the AKT signal inhibitor LY294002 (Supplementary Figure S4). As shown in Figure 6A, treatment with TGF-β1 for 2 h increased the AKT phosphorylation level, whereas chloroquine and NAC inhibited the TGF-β1-mediated induction of AKT phosphorylation. In addition, chloroquine and LY294002 treatment significantly reversed the effects of TGF-β1 on proliferation- and fibrosis-related indicators (Figure 6B). Based on the above results, we conclude that chloroquine acts as an inhibitor of the AKT signaling pathway, regulates ASM cell hypertrophy, hypersecretion and proliferation.

## Discussion

Asthmatic airway remodeling entails a wide array of pathophysiologic events such as epithelial damage, mucus gland and goblet cell hyperplasia, subepithelial fibrosis, vascular changes, and increased smooth muscle mass (including hyperplasia, parasecretion and hypertrophy) (Wang J et al., 2018). Several studies demonstrated that patients dying from severe asthma exhibited vigorous increases in the apparent smooth muscle mass within the airway wall (Ebina et al., 1993). ASM cell phenotype switching is deeply implicated in this event and favors persistent AHR and irreversible airway obstruction with a decrease in pulmonary function. ASM cells are found to be skewed towards proliferative/synthetic state in asthma and exhibit characteristics of transcription and division. Organelles associated with the synthetic phenotype predominate in the cytoplasm (Hirst, 1996). ASM cell proliferation and synthesis of cytokines or growth factors make a huge contribution to the imbalance of airway microenvironment and perpetuate the chronicity of asthma (Wang L et al., 2018). Our presented data indicated that TAS2Rs agonist chloroquine exhibited a positive effect on asthmatic mice with a significant improvement in AHR, inflammatory cell infiltration, mucus hypersecretion, collagen deposition and fibrosis. It also reduced IgE and Th2-associated cytokines, which are closely involved in the asthmatic airway microenvironment and remodeling.

As a marker of ASM cells, α-SMA is an essential component of microfilaments which connect to cell membranes (Lin et al., 1989; Colpan et al., 2019). It is pivotal for cell proliferation, hypertrophy, contraction and migration which contribute a lot to the development of airway remodeling and airway hyperresponsiveness (AHR) (Bentley and Hershenson, 2008; Xu et al., 2012; Rao et al., 2014). PCNA is also an indicator of ASM cell proliferation devoted to ASM mass (Kesavan et al., 2013; Cabral-Pacheco et al., 2020). Patients with severe asthma were confirmed to present increased levels of α-SMA and PCNA, associated with higher mortality (Kesavan et al., 2013; Zhao et al., 2013; Cabral-Pacheco et al., 2020). Our results further demonstrated that chloroquine pronouncedly downregulated α-SMA and PCNA expression within asthmatic airway walls, implying a reversion of ASM mass by chloroquine. Further study *in vitro* even showed that chloroquine suppressed TGF-β1-induced ASM cell proliferation, providing evidence for chloroquine in ASM cell hyperplasia remission in mice.

ASM cell phenotype shifts are often accompanied by a worse parasecretion state. The influx of inflammatory factors within the airways is proposed as a distinct hallmark of asthma (O’Byrne and Postma, 1999). Increased IL-8 level was observed in severe asthma (Tanabe et al., 2014). It promotes ASM cell contraction and migration (Govindaraju et al., 2006), and changes ASM cell features by eliciting the rate of cell proliferation and survival (Halwani et al., 2011). IL-8 can be secreted from ASM cells accompanied by MCP-1, ICAM-1 and VEGF (Lazaar, 2002; Rose et al., 2003; Mukhopadhyay et al., 2014). As a specific endothelial growth factor, VEGF contributes a lot to nonspecific AHR, exerts chemotactic effects on eosinophils, and enhances ASM cell proliferation (Pei et al., 2016). Previous studies showed that Th2-associated cytokines could increase VEGF production which correlated with the severity of asthma (Makinde et al., 2006). All of these suggests deteriorated airway microenvironment as a critical incentive in asthma progressing, facilitating ASM cells to the proliferative/synthetic phenotype, and aggravating a vicious circle of inflammatory response (Lazaar, 2002; Zha et al., 2013; Faiz et al., 2018). In our study, the *in vitro* findings showed that chloroquine blocked the secretion of these cytokines and adhesion molecules induced by TGF-β1 or H_2_O_2_, suggesting a novel option for airway remodeling control by rebalancing ASM cell phenotypes.

Along with this phenotype switching, the microstructure of ASM cells profoundly changed. Proliferative/synthetic ASM cells appear hypertrophy with aberrantly increased cytoplasm, organelles and microfilaments (Jones et al., 2014). Abnormal hypertrophy of ASM cells cause cell dysfunction in contraction, which determines the development and persistence of AHR in asthma (Wang J et al., 2018; Yu et al., 2020). Our TEM results revealed that TGF-β1 or H_2_O_2_ induced ASM cells to a proliferative/synthetic state, resulting in numerous cytoplasmic processes with increased profiles of organelles, such as mitochondria, highly developed Golgi cisternae and rough endoplasmic reticulum. Chloroquine markedly reversed the growth of these synthetic organelles, implying an inhibition of the high parasecretion of proliferative/synthetic ASM cells in TGF-β1/H_2_O_2_ model with the rebalance of ASM cell phenotypes.

Hyperplasia, parasecretion and hypertrophy are often present in ASM cell phenotype alterations. Other pathophysiologic events such as extracellular matrix (ECM) deposition and fibrosis involved in airway remodeling are also associated with cell phenotype switching. ECM proteins increased in asthmatic airways, particularly collagen I and fibronectin (Roche et al., 1989; Neil et al., 2007), give strong backing to ASM cell survival and proliferation. They have been proved *in vitro* to enhance the contractile signal (Johnson, 2001; Freyer et al., 2004). In turn, MMPs derived from ASM cells participated actively in ECM deposition and fibrosis (Kesavan et al., 2013; Zhao et al., 2013). Elevated levels of MMP-9 and MMP-13 were found in the serum, sputum and BALF of patients with classic asthma (Greenlee et al., 2007; Mori et al., 2012). MMP-9-deficient animals exhibited reduced airway inflammation, collagen deposition, and peribronchial fibrosis (Halade et al., 2013). Our findings indicated that chloroquine significantly restrained HDM-induced upregulation of MMP-9 and MMP-13, and substantially inhibited the expression of α-SMA, collagen I and fibronectin in ASM cells*,* sustaining that chloroquine possesses the anti-remodeling potency by controlling ASM cell phenotype. On the other hand, AKT signal inhibitor LY294002 produced the similar effects on the overexpression of α-SMA, collagen I and fibronectin induced by TGF-β1 *in vitro*, suggesting that chloroquine rebalances ASM cell phenotypes through AKT pathway.

As a serine/threonine kinase, AKT is the key mediator in phosphatidylinositol 3 kinase (PI3K)-initiated signaling closely related to ASM cell proliferation (Xu et al., 2017; Li et al., 2018). Once PI3K is activated, AKT could be phosphorylated (p-AKT) and thought as a flare of PI3K activation (Hu et al., 2019). Evidence from our previous study has suggested that ROS play key roles in AKT signal during TGF-β1 stimulation (Ma et al., 2016). ROS triggered PI3K to amplify the downstream signal, and inactivated phosphatase and tensin homolog (PTEN) which negatively regulates AKT activation (Zhang et al., 2016). It has been reported that ROS upregulated mitochondrial E3 ubiquitin protein ligase 1, induced AKT ubiquitination, and promoted proteasome degradation in head and neck cancer (Bae et al., 2012; Kim et al., 2015; Su et al., 2019). ROS-induced miRNAs modulated AKT phosphorylation to promote cellular senescence in uterine leiomyoma (Xu et al., 2018). Since AKT pathway is critical for the phenotype shifts of ASM cells, the suppression of upstream ROS may have potential implications in asthma therapy.

Our results showed that the level of *in situ* ROS was increased in the lung tissue obtained from HDM-challenged mice. And chloroquine treatment significantly attenuated the increase and presented a better benefit than DEX. *In vitro,* our data further demonstrated that chloroquine strongly inhibited the production of intracellular ROS induced by TGF-β1, and reduced the levels of MDA and IP-8. TGF-β1 and increased ROS (H_2_O_2_) directly induced ASM cell proliferation involved in airway remodeling. Chloroquine and ROS scavenger NAC notably suppressed AKT phosphorylation during the switching of ASM cell phenotype, highlighting a crucial role of ROS-AKT signaling in the phenotype reversal of ASM cells by chloroquine.

By far, the expression of TAS2Rs has been determined in multiple cell types in airway, including resident (macrophages) and migratory (neutrophils, mast cells, lymphocytes) inflammatory cells, epithelial cells, and ASM cells (Shah et al., 2009; Deshpande et al., 2011; Orsmark-Pietras et al., 2013; Ekoff et al., 2014; Maurer et al., 2015; Tran et al., 2018; Nayak et al., 2019). Activation of TAS2Rs in diverse cells may lead to distinct impacts on cell function (Lee et al., 2012; Sharma et al., 2017). As for ASM cells, a previous study has shown that chloroquine could cause their relaxation *via* TAS2Rs (Tan and Sanderson, 2014). It also plays a modulatory role in ERK1/2 phosphorylation, which were closely implicated in ASM cell proliferation (Kim et al., 2019). Besides, an antimitogenic potency of TAS2R agonists has been reported in ASM cells by inhibiting the activities of Akt kinase and S6 kinase (Sharma et al., 2016). Then our present work further provided strong evidences that chloroquine prevents ASM from the aberrant proliferation, hypersecretion and hypertrophy by reversing cellular phenotype. These findings may help to offer a potential option for the treatment of asthma, especially for the remission of airway remodeling. By using H_2_O_2_ and PI3K inhibitor (LY294002), we further confirmed that the positive effect of chloroquine may rely on the signal of ROS-AKT. The protective properties of chloroquine implicated in asthma should be studied further to determine the value and precise subtype involved in clinical application.

## Conclusion

We demonstrated the potential therapeutic action of chloroquine in asthma, revealed its properties and underlying mechanism. Collectively, our findings showed that chloroquine (I) abrogated AHR and attenuated inflammatory cell infiltration, goblet cell hyperplasia and fibrotic expression in the airways (II) decreased the overexpression of α-SMA, PCNA, MMP-9, and MMP-13 in HDM-sensitized mice (III) diminished the elevated levels of IgE, IL-4, IL-13, and TGF-β1 in the BALF (IV) reduced the *in situ* ROS in the lungs and MDA and IP-8 levels in the BALF (V) reversed TGF-β1 and H_2_O_2_-induced proliferative/synthetic ASM cells (proliferation, hypersecretion and hypertrophy); and (VI) suppressed the TGF-β1-induced production of ROS through the inhibition of AKT phosphorylation in human ASM cells. Taken together, our results indicate that chloroquine has the potential to provide beneficial improvements to AHR, airway inflammation and remodeling, and these beneficial effects are most likely due to ROS-AKT pathways. Based on these findings and its recently described bronchodilator and anti-inflammatory properties, chloroquine, as an TAS2R agonist, may be a new therapeutic approach for the treatment of allergic airway diseases.

## Data Availability

The original contributions presented in the study are included in the article/Supplementary Material, further inquiries can be directed to the corresponding authors.
