# Supplementary figures and images for: Chloroquine Attenuates Asthma Development by Restoring Airway Smooth Muscle Cell Phenotype Via the ROS-AKT Pathway
Source: Front Pharmacol. 2022 Jun 1;13:916508. doi: 10.3389/fphar.2022.916508 (PMC9198701; doi:10.3389/fphar.2022.916508)

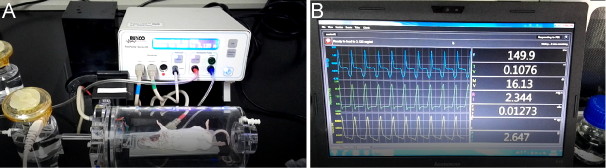

Supplement: Supplementary file 1 [file Image3.tif]

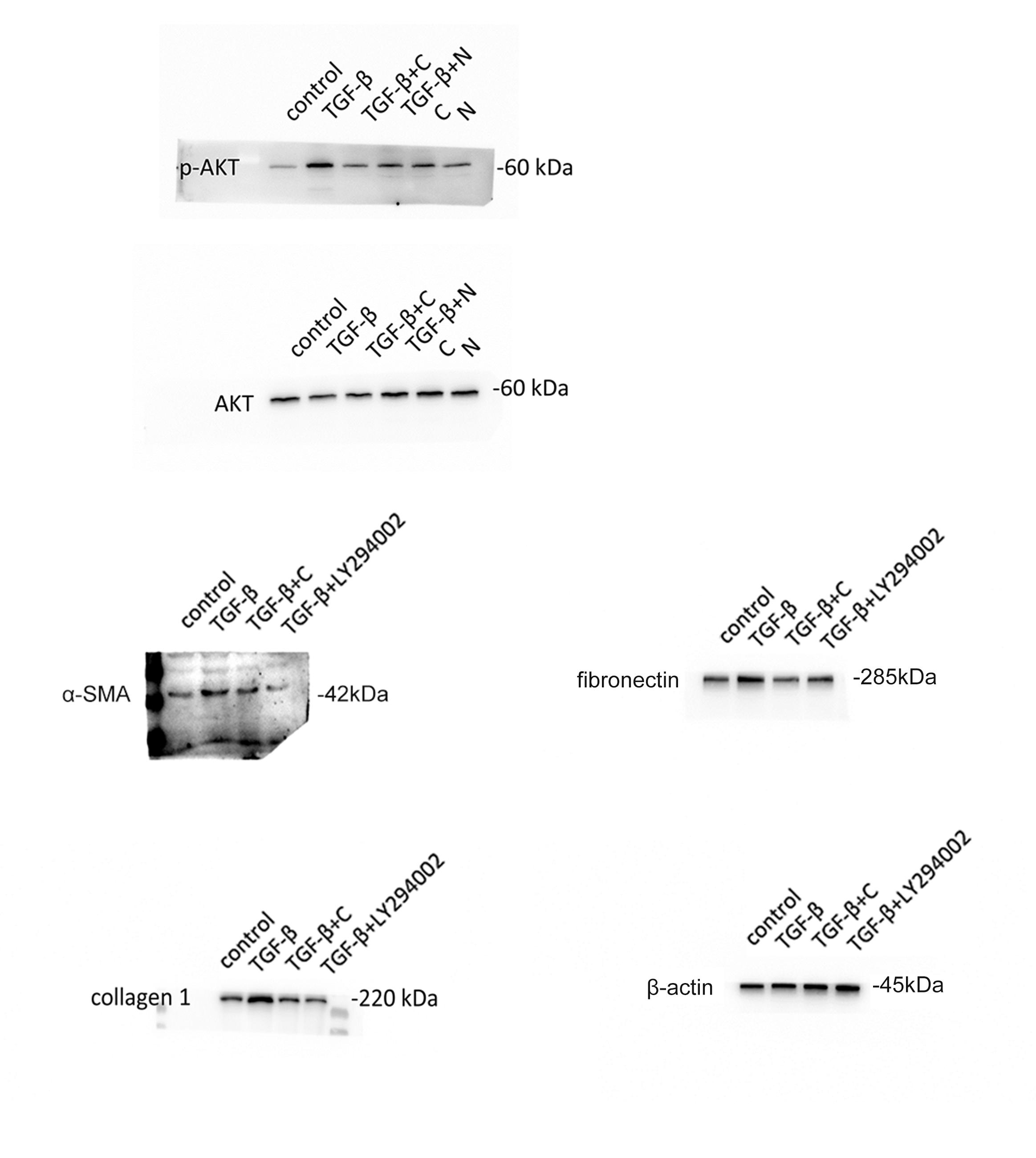

Supplement: Supplementary file 2 [file Image4.tif]

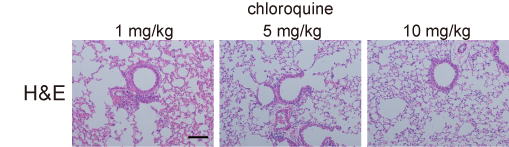

Supplement: Supplementary file 3 [file Image2.tif]

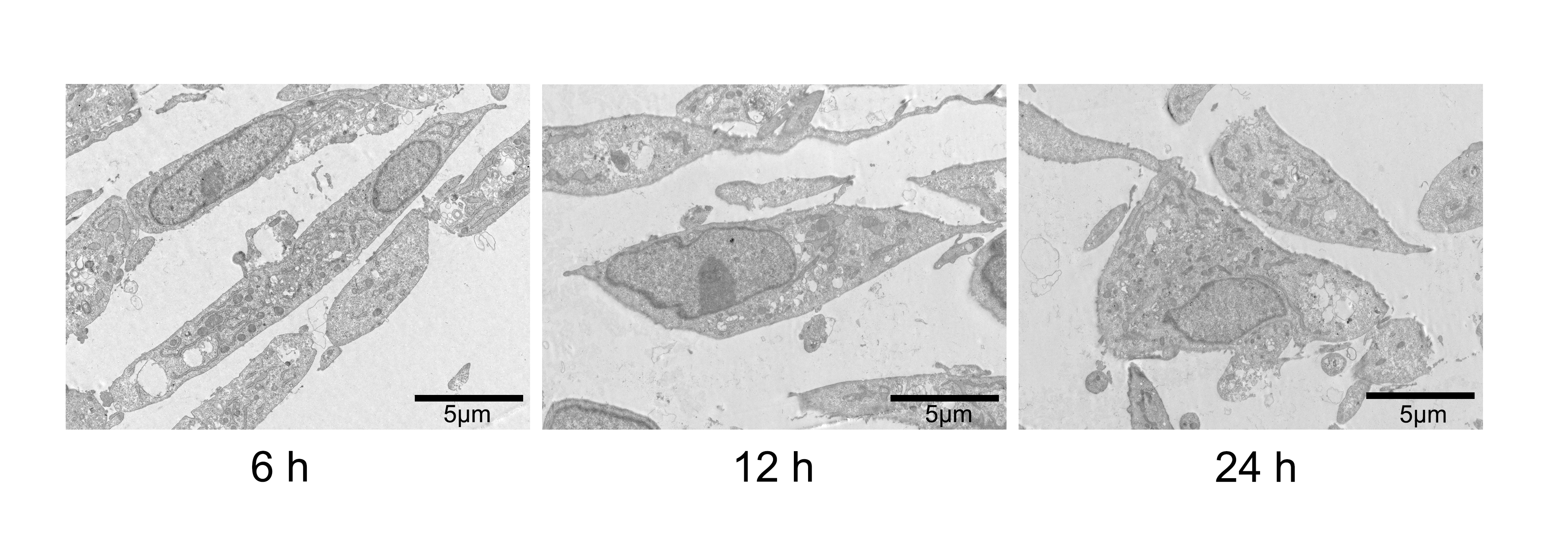

Supplement: Supplementary file 4 [file Image1.tif]
